# Psychometric Validation of the CLEFT-Q Patient Reported Outcome Measure: A Prospective Study to Examine Cross-Sectional Construct Validity

**DOI:** 10.1177/10556656211062837

**Published:** 2021-12-17

**Authors:** Anna Miroshnychenko, Charlene Rae, Karen Wong Riff, Christopher Forrest, Tim Goodacre, Marc Swan, Rona Slator, Jesse Goldstein, Achilleas Thoma, Karen Harman, Anne Klassen

**Affiliations:** 1 3710 McMaster University, Hamilton, Canada; 2 The Hospital for Sick Children, Toronto, Canada; 3 Oxford University Hospitals NHS Foundation Trust, England, UK; 4 Oxford University Hospitals NHS Foundation Trust, Spires Cleft Centre, John Radcliffe Hospital, England, UK; 5 Birmingham Children's Hospital NHS Foundation Trust, England, UK; 6 6595 University of Pittsburgh, PA (Pitt), PA, USA

**Keywords:** rhinoplasty, orthognathic surgery, bone grafting, quality of life

## Abstract

**Objective:**

CLEFT-Q is a condition-specific patient-reported outcome measure (PROM) for patients with cleft lip and/or palate (CL/P). The aim of this study was to examine the cross-sectional construct validity of the CLEFT-Q scales.

**Design:**

Construct validity was assessed through a prospective study that tested hypotheses regarding correlations of scores with other PROMs that measure related constructs.

**Setting:**

Seven cleft centres in Canada, the USA, and UK were involved.

**Patients/Participants:**

Patients were aged eight to 29 years with CL/P.

**Interventions:**

Before undergoing rhinoplasty, orthognathic, cleft lip scar revision, and alveolar bone graft, participants were asked to complete the following PROMs: CLEFT-Q (9 scales), Child Oral Health Impact Profile (socio-emotional subscale) and Cleft Hearing Appearance and Speech Questionnaire (features 1 subscale).

**Main Outcome Measure(s):**

The correlation coefficients examining the relationship between the scales were the main outcome measures. Correlations (Spearman) were calculated and interpreted as follows: <0.3 weak, 0.30 to 0.50 moderate, ≥0.50 strong.

**Results:**

Participants (*n* = 177) were mostly male (61%) and aged between eight and 11 years (42%). Overall, 38 of 52 (73%) hypotheses tested were supported. More specifically, 20 of 26 (77%) hypotheses about correlations between the appearance scales were supported, two of three (67%) hypotheses about correlations between the health-related quality of life scales were supported, and 16 of 23 (70%) hypotheses about correlations between the appearance and health-related quality of life scales were supported.

**Conclusions:**

Cross-sectional construct validity of the CLEFT-Q scales adds further evidence of the psychometric properties of this instrument.

## Introduction

Patient-reported outcome measures (PROMs) are tools designed to collect patient-reported outcomes (PROs). PROs are reports that come directly from the patients about how they function or feel in relation to a health condition and its therapy, without interpretation by a physician or anyone else (Valderas et al., 2008). There are two main types of PROMs, generic and condition-specific. Generic PROMs are instruments used in a broad range of diseases and conditions that allow for comparisons across various patient populations (Weldring and Smith, 2013). Condition-specific PROMs examine concerns that are specific to a particular condition and their impact on outcome (Monmouth Partners, 2020). The CLEFT-Q is a condition-specific PROM composed of a comprehensive set of independently functioning scales that measure appearance, health-related quality of life (HRQL) and function in individuals with a cleft lip and/or palate (CL/P).

The development of the CLEFT-Q followed a modern psychometric approach and was conducted in three phases (Wong Riff et al., 2017). The first phase consisted of identifying concepts that were important to patients with CL/P from their perspective, developing a conceptual framework based on these concepts, and creating CLEFT-Q scales to measure patients’ concerns (Tsangaris et al., 2017; Wong Riff et al., 2017). In this phase, content validity was examined, which indicatedhow relevant, comprehensive, and comprehendible the scales are to the target population (Tsangaris et al., 2017; Wong Riff et al., 2017). The second phase consisted of a field-test study that included 2434 patients from 30 sites in 12 countries, which provided evidence of construct validity, known differences between patient groups, cross-cultural validity, and internal consistency (Klassen et al., 2018). The third phase of the CLEFT-Q development aimed to examine responsiveness and further construct validity, specifically cross-sectional construct validity, in the target population. Responsiveness of the CLEFT-Q scales has been reported elsewhere (Miroshnychenko et al., in press). The focus of this article was to describe evidence of construct validity of the CLEFT-Q scales by comparing its scores with the scores of PROMs measuring similar constructs in patients with CL/P.

Criterion validity is examined by testing correlations between the newly developed measure and a measure that is accepted in the field as a “gold standard” measure for the assessment of the trait or disorder of interest (Streiner et al., 2015). A Delphi panel reached a consensus that no gold standard PROMs that measure HRQL exist (Mokkink et al., 2010a, 2010b). The consensus-based standards for the selection of health measurement instruments (COSMIN) guideline suggested that the scores of a new instrument can be compared to one or several widely used PROMs to assess construct validity, instead of criterion validity. Construct validity establishes the degree to which a PROM works as it is intended to work based on prior knowledge about the constructs being studied (Mokkink et al., 2010a, 2010b; Patrick et al., 2011a, 2011b; Streiner et al., 2015). Construct validation is a continuous process of learning about the construct, thus consists of a series of supportive studies that serve to strengthen the network of predictions of a theory (Cronbach and Meehl, 1955; Mokkink et al., 2010a, 2010b; Patrick et al., 2011a, 2011b; Streiner et al., 2015). The process of assessment of construct validity involves the following: (1) identifying the theoretical concepts and their relatedness to each other, (2) developing or identifying scales that measure these constructs, and (3) testing the correlations among these constructs (Cronbach and Meehl, 1955; Mokkink et al., 2010a, 2010b; Patrick et al., 2011a, 2011b; Streiner et al., 2015). Thus, construct validity can be assessed by testing hypotheses about the magnitude and direction of the correlation between selected instruments.

Cross-sectional construct validity is a type of construct validation that tests hypotheses about correlations between scores of measures with related constructs at a single point in time (Kirshner and Guyatt, 1985; Stucki et al., 1995; Husted et al., 2000). Construct validity of the preliminary CLEFT-Q scales was first addressed in the field-test publication (Klassen et al., 2018). The objective of this publication was to assess the cross-sectional construct validity of the CLEFT-Q scales by testing hypotheses about correlations of preoperative scores of nine CLEFT-Q scales to the scores of two other PROMs (ie, Child Oral Health Impact Profile-Short Form 19 [COHIP-SF 19] and Cleft Hearing Appearance and Speech Questionnaire [CHASQ]) commonly used to measure similar constructs in the CL/P population. Correlations amongst scales within the same top-level domains (ie, appearance and HRQL) were predicted to be strong, and correlations between scales within different top-level domains were predicted to be moderate.

## Methods

This phase 3 prospective study was conducted between January 2018 and October 2019 at seven cleft centers in Canada (The Hospital for Sick Children), the USA (Children's Hospital of Pittsburgh), and the UK (Queen Elizabeth Hospital Birmingham, Birmingham Women's and Children's Hospital, Great Ormond Street Hospital for Children, Broomfield Hospital, as well as Oxford and Salisbury Cleft Centers). Research ethics approval was attained at each participating center prior to the commencement of the study.

### Data Collection

#### The CLEFT-Q

The CLEFT-Q is a condition-specific PROM for children and young adults with CL/P (see Table 1). For this study, each patient completed a core set of CLEFT-Q scales including the appearance scales (ie, face, nose, and nostrils) and HRQL scales (ie, psychological, social, and school). Individuals undergoing the orthognathic, cleft lip scar, and alveolar bone graft (ABG) operations were provided with additional appearance scales (see Table 2). Speech function and speech distress scales were excluded, as not enough participants with an existing speech problem (*n* = 72, 40.7%) in each operation group were involved in this study (although speech outcomes may differ between centers, speech errors, especially in patients with a cleft palate, are usually corrected by eight years of age) (Nagarajan et al., 2009). Whether a participant had an existing speech problem was decided upon by their speech-language pathologist. The school scale was excluded, as it is only relevant to patients ages eight to 18 years and therefore not completed by the entire sample. The raw scale scores were converted into Rasch transformed scores ranging from 0 (worst) to 100 (best), with higher scores indicating a better outcome.

**Table 1.** Details About Each Instrument Included in the Analysis.

	CLEFT-Q	COHIP	CHASQ
Domains	(1) Appearance (2) HRQL (3) Function (excluded)	(1) Oral health (excluded) (2) Socio-emotional (3) Function (excluded)	(1) Appearance features 1 (2) Appearance features 2 (excluded)
Scales/checklists	(1) Appearance: face, lips, nose, nostrils, jaws, teeth, cleft lip scar (2) HRQL: psychological, social, school (excluded), speech distress (excluded)	Single scale	Single scale
Items	Example: Face (9 items): 1. “How much do you like…how your face looks when you look your best?” Nose (12 items): 1. “How much do you like…the length of your nose (from the top of the tip)?” Nostrils (6 items): 1. “How much do you like…how your nostrils look when you smile?” Teeth (8 items): 1. “How much do you like…the size of your teeth?” Jaws (7 items): 1. “How much do you like…the size of your jaws?” Lips (9 items): 1. “How much do you like…how your lips look when you smile?” Cleft lip scar (7 items): 1. “How much do you like…the colour of your cleft lip scar?” Psychological (10 items): 1. “I am happy with my life.” Social (10 items): 1. “My friends accept me.” School (10 items): 1. “I like seeing my friends at school.”	Socio-emotional (10 items): (1) “been unhappy or sad” (2) “felt worried or anxious” (3) “avoided smiling or laughing with other children” (4) “felt that you look different” (5) “been worried about what other people think about your …” (6) “been teased, bullied, or called names by other children” (7) missed school for any reason” (8) “not wanted to speak/read out loud in class” (9) “been confident” (10) “felt that you were attractive (good looking)”	Feature 1 (9 items): “How happy are you with: (1) how your face looks? (2) the whole of your appearance? (3) side view/profile? (4) how good-looking do you think you are?” “How do you feel about these parts of your face?: (5) nose (6) lips (7) chin (8) teeth (9) cheeks”

Abbreviations: CHASQ: Cleft Hearing Appearance and Speech Questionnaire; COHIP: Child Oral Health Impact Profile; HRQL: health-related quality of life.

**Table 2.** The CLEFT-Q Scales Included in the Assessment of Rhinoplasty, Orthognathic, Cleft lip Scar Revision, and ABG Surgeries.

	Appearance scales							HRQL scales	
	Face^a^	Nose^a^	Nostrils^a^	Teeth	Jaws	Lips	Scar	Psychological^a^	Social^a^
Rhinoplasty	Y	Y	Y					Y	Y
Orthognathic	Y	Y	Y	Y	Y	Y		Y	Y
Cleft lip scar revision	Y	Y	Y			Y	Y	Y	Y
ABG	Y	Y	Y	Y		Y		Y	Y

Abbreviations: ABG: alveolar bone graft; HRQL: health-related quality of life.

^a^ Core scales.

#### The COHIP

The COHIP-SF 19 used in this study is a short version of Child OralHhealth Impact Profile (COHIP), a PROM composed of three domains (ie, oral health, functional, and socio-emotional) that examines the impact of oral disease on quality of life (QOL) in children (see Table 1) (Broder and Wilson-Genderson, 2007). The COHIP-SF 19 socio-emotional subscale (10 items) was included and coded such that the response option “never” = 0, “almost never” = 1, “sometimes” = 2, “fairly often” = 3, and “almost all of the time” = 4 for positively worded items. The negatively worded items were reverse coded such that “never” = 4, “almost never” = 3, “sometimes” = 2, “fairly often” = 1, and “almost all of the time” = 0 (Broder and Wilson-Genderson, 2007). Total scores were computed by summing the converted scores of each item. Higher scores reflected a better outcome. Reliability and validity testing demonstrated that the COHIP-SF 19 was a psychometrically sound instrument in a school-aged pediatric population (Broder et al., 2012).

#### The CHASQ

The CHASQ is a condition-specific tool for individuals with CL/P that is a modified version of the Satisfaction with Appearance (SWA) questionnaire composed of two subscales: features 1 and features 2 (see Table 1) (Cleft Psychology Clinical Excellence Network). Features 1 subscale includes items that examine more cleft-associated features, while features 2 subscale is composed of items that assess less cleft-associated features. For the CHASQ, total scores for features 1 (9 items) were computed by adding a score of one to 10 selected by the study participant for each item (Nguyen et al., 2019). Higher scores indicated a better outcome. While SWA questionnaire and CHASQ have been used to measure outcomes in several studies, evidence addressing their psychometric properties has not yet been published (Mani et al., 2010; Feragen et al., 2015; Crerand et al., 2017; Stiernman et al., 2019, 2021).

The CLEFT-Q, COHIP, and CHASQ data were collected before and as close as possible to six months after the followining four operations: (1) rhinoplasty, (2) orthognathic, (3) cleft lip scar revision, and (4) ABG. These questionnaires were self-administered. Individuals aged eight to 29 years before undergoing any of the four cleft-related operations at any of the seven participating cleft centers were eligible. Patients with a cognitive delay were excluded. Although most sites collected data at the hospital during a clinic appointment, patient recruitment methods differed at each site based on the site's preferences and logistics (see Appendix A in the Supplemental material). All data were entered into a REDCap database hosted at the coordinating site at McMaster University, Canada (Harris et al., 2009; Harris et al., 2019). Data were downloaded from REDCap into IBM SPSS Statistics for Mac, Version 26.0, for analysis.

#### Data analysis

The COHIP socio-emotional and CHASQ features 1 subscales were included in the analysis, as these scales closely resemble constructs measured by the CLEFT-Q appearance and HRQL scales, respectively (see Table 1).

Cross-sectional construct validity was examined through testing of hypotheses about correlations of preoperative scores within and between the CLEFT-Q, COHIP, and CHASQ PROMs. Fifty-two (up to 10 hypotheses per scale) proposed hypotheses were based on the correlations between the CLEFT-Q scale scores observed in the publishedfield-test study with a sample of 2343 individuals with CL/P. Hypotheses were composed in accordance with the COSMIN recommendations (Klassen et al., 2018; Prinsen et al., 2018). Spearman correlations between a total of 11 scale scores measuring appearance or HRQL were performed to test these hypotheses. Appearance scales included seven CLEFT-Q scales (face, nose, nostrils, teeth, jaws, lips, and cleft lip scar) and the CHASQ subscale. HRQL scales included two CLEFT-Q scales (psychological and social) and the COHIP socio-emotional subscale. Since each independently functioning scale was included in up to 10 hypotheses/correlations (*n* = 10), the Bonferroni correction set the statistical significance cut-off (*P*-value) at *α*/*n* or .005.

The cross-sectional construct validity hypotheses were based on the following overall expectations: (1) correlations between appearance scales will be strong, (2) correlations between HRQL scales will be strong, and (3) correlations between appearance and HRQL scales will be moderate (see Table 3). Correlations were interpreted as follows: <0.3 weak, 0.30 to 0.50 moderate, and ≥0.50 strong (Prinsen et al., 2018). These expectations were based on the results of the field-test study, which showed that correlations between scales within the same domain were more similar than between scales in related domains (Klassen et al., 2018).

**Table 3.** Framework for Hypotheses Testing in Terms of Direction and Magnitude of Correlations Between CLEFT-Q, COHIP and CHASQ scores.

	CLEFT-Q appearance scales	CLEFT-Q HRQL scales	COHIP subscale	CHASQ subscale
	face, nose, nostrils, teeth, jaws, lips, and scar	psychological and social	socio-emotional	features 1
CLEFT-Q appearance scales	Strong correlation ≥0.5 (19 correlations)			
CLEFT-Q HRQL scales	Moderate correlation 0.3 < *x* < 0.5 (14 correlations)	Strong correlation ≥0.5 (1 correlation)		
COHIP subscale	Moderate correlation 0.3 < *x* < 0.5 (7 correlations)	Strong correlation ≥0.5 (2 correlations)		
CHASQ subscale	Strong correlation ≥0.5 (7 correlations)	Moderate correlation 0.3 < *x* < 0.5 (2 correlations)		

Abbreviations: CHASQ: Cleft Hearing Appearance and Speech Questionnaire; COHIP: Child Oral Health Impact Profile; HRQL: health-related quality of life.

## Results

Sample characteristics are shown in Table 4. A total of 177 participants were included in this phase 3 study. Most participants were from Canada and England and aged eight to 11 years. A larger proportion of participants were males (*n* = 107, 60%), students (*n* = 142, 80%), with a cleft lip and palate (*n* = 140, 79%) and without a speech problem (*n* = 95, 53.7%) or syndrome/craniofacial anomaly (*n* = 161, 91.0%).

**Table 4.** Characteristics of Participants in the CLEFT-Q Phase 3 Study.

Characteristic	No. of participants at baseline (%) *n* = 177
*Country*	
Canada	69 (39.0%)
England	70 (39.5%)
USA	38 (21.5%)
*Age, years*	
8-11	74 (41.8%)
12-15	24 (13.6%)
16-20	52 (29.4%)
≥21	27 (15.3%)
*Gender*	
Female	70 (39.5%)
Male	107 (60.5%)
*Student*	
Yes	142 (80.2%)
No	35 (19.8%)
*Cleft type*	
Cleft lip only	8 (4.5%)
Cleft palate only	3 (1.7%)
Cleft lip and palate	140 (79.1%)
Cleft lip and alveolus	24 (13.6%)
Missing	2 (1.1%)
*Current speech problem*	
No speech problem	95 (53.7%)
Mild speech problem	63 (35.6%)
Moderate speech problem	9 (5.1%)
Missing	10 (5.6%)
*Syndrome or craniofacial anomaly*	
Yes	10 (5.6%)
No	161 (91.0%)
Missing	6 (3.4%)
*Operation type*	
Rhinoplasty	38 (21.5%)
Orthognathic	27 (15.3%)
Cleft lip scar	28 (15.8%)
Alveolar bone graft (ABG)	84 (47.5%)

### Cross-Sectional Construct Validity

The analysis to examine cross-sectional construct validity included a sample of 177 participants. Spearman correlations and the number of participants included in each analysis are shown in Table 5. Correlations between the cleft lip scar scale and the jaws and teeth scales were not possible, as no participant who completed the cleft lip scar scale also completed either the jaws or teeth scale. Of 52 correlations, 38 (73%) aligned with the predetermined hypotheses. The findings are described in more detail below.

**Table 5.** A total of 52 Correlations of Preoperative Scores of Patients Undergoing Rhinoplasty, Orthognathic, Cleft lip Scar Revision and ABG Surgeries Were Examined.

		CLEFT-Q appearance scales							CLEFT-Q HRQL scales		COHIP	CHASQ
		Face	Jaws	Lips	Nose	Nostrils	Scar	Teeth	Psychological	Social	Socio-emotional	Features 1
Face	*r*	1										
	*n*	177										
Jaws	*r*	0.75**	1									
	*n*	25	25									
Lips	*r*	0.71**	0.67**	1								
	*n*	135	25	135								
Nose	*r*	0.63**	0.57**	0.60**	1							
	*n*	175	25	134	175							
Nostrils	*r*	0.60**	0.53**	0.59**	0.62**	1						
	*n*	174	25	135	173	174						
Scar	*r*	0.32*	—	0.56**	0.38*	0.24	1					
	*n*	28	0	28	28	28	28					
Teeth	*r*	0.56**	0.70**	0.56**	0.39**	0.45**	—	1				
	*n*	108	25	107	107	108	0	108				
Psychological	*r*	0.53**	0.41**	0.44**	0.39**	0.35**	0.18	0.29**	1			
	*n*	173	24	134	172	173	28	107	173			
Social	*r*	0.47**	0.37*	0.38**	0.33**	0.35**	0.11	0.33**	0.66**	1		
	*n*	172	24	133	171	172	28	106	172	172		
COHIP	*r*	0.47**	0.39**	0.42**	0.35**	0.28**	0.34*	0.39**	0.44**	0.55**	1	
	*n*	165	24	127	164	165	28	100	165	164	165	
CHASQ	*r*	0.67**	0.73**	0.67**	0.56**	0.52**	0.60**	0.46**	0.57**	0.50**	—	1
	*n*	165	24	128	164	165	27	102	165	164	—	165

**Abbreviations:** ABG: alveolar bone graft; CHASQ: Cleft Hearing Appearance and Speech Questionnaire; COHIP: Child Oral Health Impact Profile; HRQL: health-related quality of life. **P < .005.

#### Correlations between appearance scales

Correlations between the appearance scales were expected to be strong (*r* ≥ 0.50). A total of 26 correlations were performed to compare the eight appearance scales. Of the total 26 hypotheses, 20 (71%) were supported by the results. Six of the seven hypotheses to examine correlations between the CHASQ subscale and CLEFT-Q appearance scales were supported (*r* ≥ 0.5, *P* = 0.005). The exception was the correlation between the CHASQ subscale and the CLEFT-Q teeth scale, which was slightly weaker than predicted (*r* = 0.46, *P* = 0.005). Fourteen of 19 hypotheses testing correlations among the CLEFT-Q appearance scales were supported (*r* ≥ 0.5, *P* =  0.005). Of the remaining five correlations, four (face and scar, nose and scar, nose and teeth, and nostrils and teeth) were moderate (0.3 < *r* < 0.5, *P* = 0.005) and one (nostrils and scar) was weak (*r* < 0 .3, *P* = 0.005).

#### Correlations between HRQL scales

Correlations between the three HRQL scales were expected to be strong (*r* ≥ 0.50). Of the three hypotheses tested, two were supported by the study results. The hypotheses comparing the two CLEFT-Q scales (psychological and social), and the COHIP subscale and CLEFT-Q social scale were supported (*r* ≥ 0.5, *P* = 0.005). The correlation between the COHIP subscale and CLEFT-Q psychological scale was slightly lower than predicted (0.3 < *r* < 0.5, *P* = 0.005).

#### Correlations between appearance and HRQL scales

Correlations between the appearance and HRQL scales were expected to be moderate (0.3 < *r* < 0.5). Of the total 23 hypotheses, 16 (70%) were supported by the study results. In the correlations between the CLEFT-Q scales, 10 of 14 hypotheses to evaluate correlations between the CLEFT-Q appearance and both the CLEFT-Q psychological and social scales were supported by the results (0.3 < *r* < 0 .5, *P* = 0.005). Of the four hypotheses that were not supported, three were correlations between the HRQL scales and both the cleft lip scar and teeth scales, which were weaker than expected (0.3 > *r*, *P* = 0.005). The remaining of the 4 was a correlation between the psychological and face scales, which was slightly stronger than expected (*r* ≥ 0.5, *P* = 0.005).

In the analyses between the CLEFT-Q appearance scales and COHIP subscales, six of seven hypotheses were supported (0.3 < *r* < 0.5, *P* = 0.005). The exception was a slightly weaker than the predicted correlation between the nostrils scale and COHIP subscale (0.3 > *r*, *P* = 0.005).

In the analyses between the CLEFT-Q psychological and social scales and CHASQ subscale, neither hypothesis was supported; the correlations were slightly stronger than expected (*r* ≥ 0.5, *P* = 0.005).

## Discussion

The CLEFT-Q scales comprise a condition-specific PROM for patients with CL/P. The CLEFT-Q scales have been shown to have a positive impact onthe way patients feel about their appearance (Klassen et al., 2020). The positive impact that the CLEFT-Q scales have on children and young adults who complete them (Klassen et al, 2020), as well as their treatment and outcomes may have contributed to its rapid uptake by clinicians and academics worldwide. Assessment of the psychometric properties of CLEFT-Q scales, such as construct validity, is essential for establishing its use in research and clinical setting, as evidence of construct validity signals that an instrument is measuring the constructs that it was designed to measure. Assessment of cross-sectional construct validity consisted of testing whether the scores collected at the study baseline corresponded with the theoretical expectations based on the results of the second phase international field-test study (Klassen et al., 2018). In the field-test study, correlations between scales within the same domain were more similar than between scales in related domains. Therefore, correlations amongst the scales within their top-level domains (ie, appearance and HRQL) were predicted to be strong and between scales in different top-level domains were predicted to be moderate. The results of this study indicate cross-sectional construct validity of the CLEFT-Q scales, thus adding to the existing body of evidence that supports its psychometric properties (Tsangaris et al., 2017; Klassen et al., 2018; Harrison et al., 2019).

Of 52 correlations to examine relationships between CLEFT-Q scales and CHASQ and COHIP subscales, 38 (73%) aligned with the predetermined hypotheses. Of 14 hypotheses that were not supported, 11 were weaker than anticipated and three were stronger. Eight of these 14 correlations were exceptionally close to the prediction, while six were not. Five of these six correlations compared the CLEFT-Q appearance (face, nose, and nostrils) and HRQL (psych and social) with the CLEFT-Q cleft lip scar scale. The cleft lip scar scale sample size was smaller than anticipated (*n* = 28), which may explain the correlation coefficients being lower than expected. The remaining correlation compared the CLEFT-Q teeth and nose scales. This correlation may not have reached its prediction due to the teeth scale being administered only to individuals undergoing operations involving their gums, that is, orthognathic and ABG, which do not directly affect the nose.

The findings for cross-sectional construct validity in this study add to the published evidence about construct validity from the field-test study sample. Specifically, mean scores from 1938 patients who needed, had, and did not require rhinoplasty, orthognathic, cleft lip scar revision, and speech surgeries were published (Harrison et al., 2019). The authors reported that participants who needed surgery scored significantly lower than those who had surgery on CLEFT-Q scales relevant to each surgery. These results suggest that the CLEFT-Q scales were able to detect differences between groups cross-sectionally based on surgical status (Harrison et al., 2019).

Although most psychometric qualities of CLEFT-Q scales have been examined, several have yet to be assessed in accordance with COSMIN recommendations (Prinsen et al., 2018). COSMIN gold standard of assessing PROMs covers validity, reliability, and responsiveness psychometric qualities. To date, the following components of validity of the CLEFT-Q scales have been addressed: content validity, structural validity, and cross-cultural validity (Tsangaris et al., 2017; Klassen et al., 2018). Further evidence of cross-sectional construct validity was demonstrated in this publication, with 38 of 52 (73%) hypotheses supported by the study results. Future work to assess longitudinal construct validity is required. To examine the reliability, internal consistency, a component of reliability, has been tested in the second phase of CLEFT-Q development (Klassen et al., 2018). However, reproducibility and measurement error, other elements of reliability, have not been addressed. The CLEFT-Q scales were designed to be responsive to cleft-related treatment, therefore assessment of responsiveness is required. Evidence of external responsiveness is the focus of a separate publication (Miroshnychenko et al., in press).

The process of developing the CLEFT-Q has been a multidisciplinary and multisite initiative with partners around the globe. Collaborating with international teams ensured that the rigorous development and validation processes account for multicultural perspectives on cleft-related care. The swift uptake of CLEFT-Q scales in 45 countries, and its translation into 22 languages as of November 2021 is evidence of its useful, comprehensive, and relevant nature. Inclusion of the CLEFT-Q scales in the International Consortium for Health Outcome Measurement cleft standard set provides a means for hospitals worldwide to adopt the scales for use in clinical practice, with potential for global benchmarking (Allori et al., 2017). Evidence of cross-sectional construct validity of the CLEFT-Q scales further supports the validity of this instrument and its use for research and clinical care.

### Limitations

A limitation of this study was a small sample size for CLEFT-Q cleft lip scar (*n* = 27) and jaws (*n* = 24) scales that may have limited our ability to precisely examine cross-sectional construct validity for these scales. The version of the COHIP provided by the developer to our research team was missing a school-related item (#8) from the socio-emotional subscale (see Table 1). The mean of the remaining items was imputed for this item to score the scale. Further, this COHIP version had one school-related question. Participants who were not attending school were asked to think of another social institution they attend when answering this question.

Another limitation was that the age range of individuals included in this study was slightly broader than the suggested age range for use of COHIP and CHASQ (ie, 7-18 and 10-20 years, respectively). A broader age range was permitted to include all participants who demonstrated a strong interest in participating in the study, but who, nonetheless, were able to independently read, understand, and answer all items of all three questionnaires. The validity and reliability of the CHASQ scale have not been published in a peer-reviewed journal, thereby further limiting the results. Additionally, the sample size for the CLEFT-Q school, speech distress and speech function scales were too small to include these scales in the analysis. The generalizability of the study results may be limited, given it was conducted in only three countries, that is, the US, Canada, and the UK. Assessment of cross-sectional construct validity using data collected in other countries will be beneficial. Further research could also investigate the cross-sectional construct validity of the CLEFT-Q school scale by comparing its scores to the COHIP socio-emotional subscale, as well as comparing the CLEFT-Q speech distress and speech function scales with scores form the COHIP functional subscale.

## Conclusion

The CLEFT-Q is a rigorously developed PROM for individuals with CL/P and its psychometric properties have been tested throughout its 3-phase development process. In the present study, assessment of correlations between the CLEFT-Q scales and COHIP and CHASQ subscales supported most prespecified hypotheses, thus providing strong evidence for the cross-sectional construct validity of the CLEFT-Q scales. Further examination of longitudinal construct validity is required.

## Supplemental Material

sj-docx-1-cpc-10.1177_10556656211062837 - Supplemental material for Psychometric Validation of the CLEFT-Q Patient Reported Outcome Measure: A Prospective Study to Examine Cross-Sectional Construct Validity Click here for additional data file. Supplemental material, sj-docx-1-cpc-10.1177_10556656211062837 for Psychometric Validation of the CLEFT-Q Patient Reported Outcome Measure: A Prospective Study to Examine Cross-Sectional Construct Validity by Anna Miroshnychenko, Charlene Rae, Karen Wong Riff, Christopher Forrest, Tim Goodacre, Marc Swan, Rona Slator, Jesse Goldstein, Achilleas Thoma, Karen Harman and Anne Klassen in The Cleft Palate Craniofacial Journal
