# Supplementary material for: Psychometric Validation of the CLEFT-Q Patient Reported Outcome Measure: A Prospective Study to Examine Cross-Sectional Construct Validity
Source: Cleft Palate Craniofac J. 2021 Dec 17;60(3):327–35. doi: 10.1177/10556656211062837 (PMC9900191; doi:10.1177/10556656211062837)
Supplement: sj-docx-1-cpc-10.1177_10556656211062837 - Supplemental material for Psychometric Validation of the CLEFT-Q Patient Reported Outcome Measure: A Prospective Study to Examine Cross-Sectional Construct Validity [file sj-docx-1-cpc-10.1177_10556656211062837.docx]

**Appendix A**

| Recruitment methodology before and after operation at each site. | | | | | |
| --- | --- | --- | --- | --- | --- |
| Site | Recruiter | Location (preop) | Location (postop) | Data collection | Data collection |
| The Hospital for Sick Children | Researcher | Hospital | Home | Tablet | Electronic |
| Children’s Hospital of Pittsburgh | Researcher | Clinic/home | Clinic/home | Paper | Paper |
| Broomfield Hospital | Research nurse | Clinic | Clinic | Paper | Paper |
| University Hospitals Birmingham | Research nurse | Clinic | Clinic | Paper | Paper |
| Great Ormond Street Hospital | Psychologist | Clinic | Clinic | Paper | Paper |
| Oxford & Salisbury Cleft Centers | Research nurse | Clinic | Clinic | Paper | Paper |
